# 1,1′-(4,4′-Bipiperidine-1,1′-di­yl)bis­(2,2,2-trifluoro­ethanone)

**DOI:** 10.1107/S1600536811022434

**Published:** 2011-06-18

**Authors:** Vitthal N. Yadav, Tore Hansen, Carl Henrik Görbitz

**Affiliations:** a Department of Chemistry, University of Oslo, Oslo, Norway

## Abstract

The title compound, C_14_H_18_F_6_N_2_O_2_, has a central center of symmetry with both piperidine rings occurring in regular chair conformations. Even though the structure is fairly compact with no sizable voids, the shortest H⋯O distance is as long as 2.58 Å.

## Related literature

For applications of and structures related to 4,4′-bipiperidine compounds, see: Medina *et al.* (1991 ▶); Li *et al.* (2009 ▶); Wang *et al.* (2007 ▶); Melchiorre *et al.* (2001 ▶); Adams *et al.* (2006 ▶); Angeloni & Orpen (2001 ▶); De las Casas Engel *et al.* (2010 ▶). For a related synthesis, see: Schenck *et al.* (2004 ▶). For inter­pretation of C—H⋯F bond configurations, see: Shimoni & Glusker (1994 ▶). For the use of a large specimen for data collection, see: Görbitz (1999 ▶).
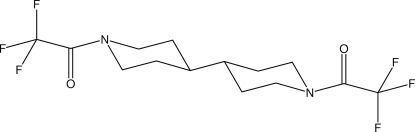


## Experimental

### 

#### Crystal data

C_14_H_18_F_6_N_2_O_2_
*M* *_r_* = 360.30
Triclinic, 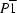

*a* = 6.6825 (12) Å
*b* = 6.7350 (12) Å
*c* = 9.3089 (16) Å
α = 99.952 (2)°
β = 108.564 (2)°
γ = 101.542 (2)°
*V* = 376.30 (12) Å^3^
*Z* = 1
Mo *K*α radiation
μ = 0.16 mm^−1^
*T* = 105 K
1.00 × 0.50 × 0.25 mm

#### Data collection

Bruker APEXII CCD diffractometer
Absorption correction: multi-scan (*SADABS*; Bruker, 2007 ▶) *T*_min_ = 0.921, *T*_max_ = 0.962
3303 measured reflections
1731 independent reflections
1586 reflections with *I* > 2σ(*I*)
*R*_int_ = 0.009

#### Refinement

*R*[*F* ^2^ > 2σ(*F* ^2^)] = 0.029
*wR*(*F* ^2^) = 0.080
*S* = 1.06
1731 reflections
109 parameters
H-atom parameters constrained
Δρ_max_ = 0.39 e Å^−3^
Δρ_min_ = −0.23 e Å^−3^

### 

Data collection: *APEX2* (Bruker, 2007 ▶); cell refinement: *SAINT-Plus* (Bruker, 2007 ▶); data reduction: *SAINT-Plus*; program(s) used to solve structure: *SHELXTL* (Sheldrick, 2008 ▶); program(s) used to refine structure: *SHELXTL*; molecular graphics: *SHELXTL*; software used to prepare material for publication: *SHELXTL*.

## Supplementary Material

Crystal structure: contains datablock(s) I, global. DOI: 10.1107/S1600536811022434/ng5179sup1.cif

Structure factors: contains datablock(s) I. DOI: 10.1107/S1600536811022434/ng5179Isup2.hkl

Supplementary material file. DOI: 10.1107/S1600536811022434/ng5179Isup3.cml

Additional supplementary materials:  crystallographic information; 3D view; checkCIF report

## Figures and Tables

**Fig. 1.**
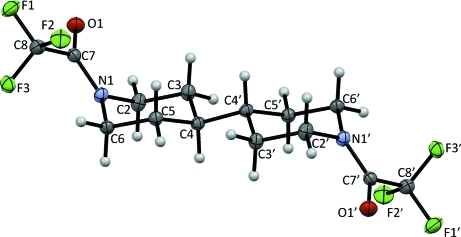
The structure of (I), showing the atom-labelling scheme. Displacement ellipsoids are drawn at the 50% probability level; H atoms are spheres of arbitrary size.

**Fig. 2.**
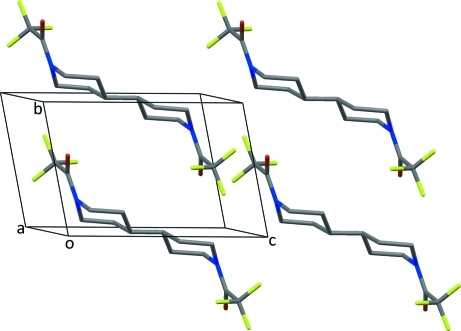
The unit cell and three-dimensional crystal packing of (I) viewed approximately along the a-axis. Hydrogen atoms have been left out for clarity.

## supplementary crystallographic information

### Comment

The structures containing 4,4'-bipiperidine scaffold attract more interest as molecular spacer for sustaining functional diversity, which finds different applications in materials chemistry (Wang *et al.*, 2007; Medina *et al*., 1991; Li *et al.*, 2009; Adams *et al.*, 2006; Angeloni & Orpen, 2001), Organocatalysis (De las Casas Engel *et al.*, 2010) and pharmaceutical development (Melchiorre *et al.*, 2001).

The asymmetric unit contain one half of the *N*,*N*'-di-trifluoroacetyl-4,4'-bipiperidine molecule. The molecular structure is shown in Fig. 1. The intermolecular interactions between –C—H···F–, –F···F–, C=O···F–, C—H ··· O=C and π ···O=C leading to the supramolecular three-dimensional crystal packing.

In the significant –C—H···F– interactions, the fluorine atom acts as an H-bond acceptor. The distances between –H···F– nearly 2.572 Å shows important and binding interactions are mainly electrostatic. The angles –C—H···F-(169.03 & 147.63 °) are linear, indicates repulsive interactions (Shimoni & Glusker, 1994).

### Experimental

The compound (I) was prepared by the procedure reported for piperidine by Schenck *et al.* (2004). The 4,4'-bipiperidyl dihydrochloride (0.5 g, 2.07 mmol) and triethyl amine (1.2 ml, 8.6 mmol) mixed together at 0 °C in 20 ml of dry diethyl ether, stirred the mixture for 10 min. and added trifluoro acetic anhydride (0.61 ml, 4.3 mmol) dropwise, continued stirring at room temperature for 3 h., added 1 ml of 2*M* HCl and stirred for 10 min., filtered the solid residue and washed with fresh diethyl ether.

The highly stable X-ray quality crystals were obtained by slow evaporation of dichloromethane. A rather large specimen (maximum dimension 1.00 mm) was used for data collection to get high diffraction intensitites. Previous investigations indicate that this does not represent a problem for a light-atom-only structure (see Görbitz, 1999).

### Refinement

The hydrogen atoms were placed at calculated position with C—H = 0.99 Å for CH_2_ and 1.00 Å for CH. For all H atoms *U*_iso_(H) values were fixed at 1.2*U*_eq_ of the carrier atom.

### Crystal data

**Table:**

C_14_H_18_F_6_N_2_O_2_	*Z* = 1
*M**_r_* = 360.30	*F*(000) = 186
Triclinic, *P*1	*D*_x_ = 1.590 Mg m^−^^3^
Hall symbol: -P 1	Melting point: 397 K
*a* = 6.6825 (12) Å	Mo *K*α radiation, λ = 0.71073 Å
*b* = 6.7350 (12) Å	Cell parameters from 2545 reflections
*c* = 9.3089 (16) Å	θ = 2.4–28.3°
α = 99.952 (2)°	µ = 0.16 mm^−^^1^
β = 108.564 (2)°	*T* = 105 K
γ = 101.542 (2)°	Rods, colourless
*V* = 376.30 (12) Å^3^	1.00 × 0.50 × 0.25 mm

### Data collection

**Table:**

Bruker APEXII CCD diffractometer	1731 independent reflections
Radiation source: fine-focus sealed tube	1586 reflections with *I* > 2σ(*I*)
graphite	*R*_int_ = 0.009
Detector resolution: 8.3 pixels mm^-1^	θ_max_ = 28.6°, θ_min_ = 2.4°
Sets of exposures each taken over 0.5° ω rotation scans	*h* = −8→8
Absorption correction: multi-scan (*SADABS*; Bruker, 2007)	*k* = −8→8
*T*_min_ = 0.921, *T*_max_ = 0.962	*l* = −12→12
3303 measured reflections	

### Refinement

**Table:**

Refinement on *F*^2^	Primary atom site location: structure-invariant direct methods
Least-squares matrix: full	Secondary atom site location: difference Fourier map
*R*[*F*^2^ > 2σ(*F*^2^)] = 0.029	Hydrogen site location: inferred from neighbouring sites
*wR*(*F*^2^) = 0.080	H-atom parameters constrained
*S* = 1.06	*w* = 1/[σ^2^(*F*_o_^2^) + (0.0398*P*)^2^ + 0.1258*P*] where *P* = (*F*_o_^2^ + 2*F*_c_^2^)/3
1731 reflections	(Δ/σ)_max_ = 0.008
109 parameters	Δρ_max_ = 0.39 e Å^−^^3^
0 restraints	Δρ_min_ = −0.23 e Å^−^^3^

### Special details

**Table:**

Geometry. All e.s.d.'s (except the e.s.d. in the dihedral angle between two l.s. planes) are estimated using the full covariance matrix. The cell e.s.d.'s are taken into account individually in the estimation of e.s.d.'s in distances, angles and torsion angles; correlations between e.s.d.'s in cell parameters are only used when they are defined by crystal symmetry. An approximate (isotropic) treatment of cell e.s.d.'s is used for estimating e.s.d.'s involving l.s. planes.
Refinement. Refinement of *F*^2^ against ALL reflections. The weighted *R*-factor *wR* and goodness of fit *S* are based on *F*^2^, conventional *R*-factors *R* are based on *F*, with *F* set to zero for negative *F*^2^. The threshold expression of *F*^2^ > σ(*F*^2^) is used only for calculating *R*-factors(gt) *etc*. and is not relevant to the choice of reflections for refinement. *R*-factors based on *F*^2^ are statistically about twice as large as those based on *F*, and *R*- factors based on ALL data will be even larger.

### Fractional atomic coordinates and isotropic or equivalent isotropic displacement parameters (Å^2^)

**Table:**

	*x*	*y*	*z*	*U*_iso_*/*U*_eq_	
F1	0.11060 (12)	0.31663 (10)	0.84127 (9)	0.03113 (19)	
F2	0.00855 (11)	0.51373 (11)	0.68885 (8)	0.02834 (18)	
F3	0.13374 (10)	0.63747 (10)	0.93992 (7)	0.02236 (16)	
O1	0.49112 (14)	0.43556 (12)	0.82275 (10)	0.02475 (19)	
N1	0.47091 (14)	0.76768 (13)	0.81595 (10)	0.01700 (19)	
C2	0.68525 (17)	0.82626 (17)	0.80093 (12)	0.0199 (2)	
H21	0.7640	0.7193	0.8255	0.024*	
H22	0.7745	0.9622	0.8764	0.024*	
C3	0.65544 (17)	0.84357 (16)	0.63442 (12)	0.0186 (2)	
H31	0.5798	0.7036	0.5607	0.022*	
H32	0.8014	0.8906	0.6274	0.022*	
C4	0.52192 (16)	0.99793 (15)	0.58605 (11)	0.0155 (2)	
H41	0.6099	1.1413	0.6534	0.019*	
C5	0.30751 (16)	0.94359 (15)	0.61687 (11)	0.0166 (2)	
H51	0.2321	1.0545	0.5987	0.020*	
H52	0.2099	0.8101	0.5418	0.020*	
C6	0.34705 (17)	0.92258 (15)	0.78370 (12)	0.0171 (2)	
H61	0.4304	1.0600	0.8592	0.021*	
H62	0.2047	0.8779	0.7965	0.021*	
C7	0.39316 (17)	0.57031 (16)	0.82058 (11)	0.0176 (2)	
C8	0.15978 (18)	0.51030 (16)	0.82412 (13)	0.0210 (2)	

### Atomic displacement parameters (Å^2^)

**Table:**

	*U*^11^	*U*^22^	*U*^33^	*U*^12^	*U*^13^	*U*^23^
F1	0.0357 (4)	0.0179 (3)	0.0457 (4)	0.0046 (3)	0.0226 (3)	0.0114 (3)
F2	0.0223 (3)	0.0307 (4)	0.0252 (3)	0.0026 (3)	0.0040 (3)	0.0043 (3)
F3	0.0247 (3)	0.0240 (3)	0.0250 (3)	0.0098 (3)	0.0148 (3)	0.0082 (3)
O1	0.0318 (4)	0.0201 (4)	0.0305 (4)	0.0140 (3)	0.0164 (4)	0.0093 (3)
N1	0.0180 (4)	0.0177 (4)	0.0194 (4)	0.0078 (3)	0.0087 (3)	0.0080 (3)
C2	0.0169 (5)	0.0246 (5)	0.0218 (5)	0.0080 (4)	0.0078 (4)	0.0110 (4)
C3	0.0184 (5)	0.0217 (5)	0.0207 (5)	0.0093 (4)	0.0093 (4)	0.0095 (4)
C4	0.0164 (4)	0.0152 (4)	0.0163 (5)	0.0055 (4)	0.0065 (4)	0.0052 (4)
C5	0.0175 (5)	0.0175 (4)	0.0174 (5)	0.0073 (4)	0.0072 (4)	0.0063 (4)
C6	0.0211 (5)	0.0160 (4)	0.0185 (5)	0.0090 (4)	0.0093 (4)	0.0065 (4)
C7	0.0218 (5)	0.0174 (5)	0.0157 (4)	0.0070 (4)	0.0086 (4)	0.0043 (4)
C8	0.0238 (5)	0.0171 (5)	0.0233 (5)	0.0053 (4)	0.0103 (4)	0.0058 (4)

### Geometric parameters (Å, °)

**Table:**

F1—C8	1.3299 (12)	C3—H31	0.9900
F2—C8	1.3478 (13)	C3—H32	0.9900
F3—C8	1.3363 (12)	C4—C5	1.5354 (14)
O1—C7	1.2199 (13)	C4—C4^i^	1.5404 (19)
N1—C7	1.3403 (13)	C4—H41	1.0000
N1—C2	1.4654 (13)	C5—C6	1.5268 (13)
N1—C6	1.4694 (12)	C5—H51	0.9900
C2—C3	1.5267 (14)	C5—H52	0.9900
C2—H21	0.9900	C6—H61	0.9900
C2—H22	0.9900	C6—H62	0.9900
C3—C4	1.5352 (13)	C7—C8	1.5433 (15)
			
C7—N1—C2	118.28 (8)	C6—C5—C4	112.24 (8)
C7—N1—C6	127.41 (9)	C6—C5—H51	109.2
C2—N1—C6	112.38 (8)	C4—C5—H51	109.2
N1—C2—C3	110.03 (8)	C6—C5—H52	109.2
N1—C2—H21	109.7	C4—C5—H52	109.2
C3—C2—H21	109.7	H51—C5—H52	107.9
N1—C2—H22	109.7	N1—C6—C5	109.91 (8)
C3—C2—H22	109.7	N1—C6—H61	109.7
H21—C2—H22	108.2	C5—C6—H61	109.7
C2—C3—C4	111.99 (8)	N1—C6—H62	109.7
C2—C3—H31	109.2	C5—C6—H62	109.7
C4—C3—H31	109.2	H61—C6—H62	108.2
C2—C3—H32	109.2	O1—C7—N1	125.64 (10)
C4—C3—H32	109.2	O1—C7—C8	117.66 (9)
H31—C3—H32	107.9	N1—C7—C8	116.70 (9)
C5—C4—C3	109.77 (8)	F1—C8—F3	107.55 (8)
C5—C4—C4^i^	111.59 (10)	F1—C8—F2	107.07 (9)
C3—C4—C4^i^	111.60 (10)	F3—C8—F2	107.07 (9)
C5—C4—H41	107.9	F1—C8—C7	110.32 (9)
C3—C4—H41	107.9	F3—C8—C7	113.47 (9)
C4^i^—C4—H41	107.9	F2—C8—C7	111.08 (8)
			
C7—N1—C2—C3	104.98 (10)	C2—N1—C7—O1	4.65 (15)
C6—N1—C2—C3	−60.38 (11)	C6—N1—C7—O1	167.54 (10)
N1—C2—C3—C4	55.87 (11)	C2—N1—C7—C8	−175.62 (8)
C2—C3—C4—C5	−51.51 (11)	C6—N1—C7—C8	−12.72 (15)
C2—C3—C4—C4^i^	−175.77 (10)	O1—C7—C8—F1	4.75 (13)
C3—C4—C5—C6	51.41 (11)	N1—C7—C8—F1	−175.01 (8)
C4^i^—C4—C5—C6	175.67 (9)	O1—C7—C8—F3	125.51 (10)
C7—N1—C6—C5	−103.67 (11)	N1—C7—C8—F3	−54.25 (12)
C2—N1—C6—C5	60.06 (11)	O1—C7—C8—F2	−113.81 (10)
C4—C5—C6—N1	−55.39 (11)	N1—C7—C8—F2	66.43 (12)

Symmetry codes: (i) −*x*+1, −*y*+2, −*z*+1.
